# Coronavirus disease 2019 drug discovery through molecular docking

**DOI:** 10.12688/f1000research.24218.1

**Published:** 2020-06-03

**Authors:** Sweta Singh, Hector Florez

**Affiliations:** 1 Savitribai Phule Pune University, Pune, India; 2 Universidad Distrital Francisco Jose de Caldas, Bogota, Colombia

**Keywords:** COVID-19, SARS-CoV-2, Molecular Docking

## Abstract

**Background: **The dawn of the year 2020 witnessed the spread of the highly infectious and communicable disease coronavirus disease 2019 (COVID-19) globally since it was ﬁrst reported in 2019. Severe acute respiratory syndrome coronavirus-2 is the main causative agent. In total, 3,096,626 cases and 217,896 deaths owing to COVID-19 were reported by 30th April, 2020 by the World Health Organization. This means infection and deaths show an exponential growth globally. In order to tackle this pandemic, it is necessary to ﬁnd possible easily accessible therapeutic agents till an effective vaccine is developed.

**Methods:** In this study, we present the results of molecular docking processes through high throughput virtual screening to analyze drugs recommended for the treatment of COVID-19.

**Results:** Atovaquone, fexofenadine acetate (Allegra), ethamidindole, baicalin, glycyrrhetic acid, justicidin D, euphol, and curine are few of the lead molecules found after docking 129 known antivirals, antimalarial, antiparasitic drugs and 992 natural products.

**Conclusions: **These molecules could act as an effective inhibitory drug against COVID-19.

## Introduction

Coronavirus Disease 2019 (COVID-19) is caused by the severe acute respiratory syndrome coronavirus-2 (SARS-CoV-2), that is responsible for respiratory illness and probably many more is yet to be discovered. This novel virus was first identified on 30 ^th^ December, 2019, with its first infection case infecting a human, which was reported in Wuhan city located in Hubei, China ^1^. Coronaviruses are mainly zoonotic, and are present amongst birds and mammals, causing respiratory, neurological, hepatic and enteric diseases ^2^ as well as comprises of enveloped RNA. The World Health Organization (WHO) declared this disease as a pandemic on 11th March, 2020 and SARS-CoV-2 as the deadliest virus till date on earth claiming 217,896 deaths till 30 ^th^ April, 2020 ^3^.

Then, it is necessary for the rapid development and approval of a vaccine, which is not yet available ^4^. Nevertheless, Chang *et al*., ^5^ have suggested that some drugs against same type of viruses approved by the US Food and Drug Administration (FDA) might offer promising results. Hydroxychloroquine is one such drug that is used worldwide whereas Remdesivir and Ivermectin have been reported to work against COVID-19 *in silico* by others.

The transmission of this coronavirus occurs due to the binding of the CoV spike protein to the angiotensin converting enzyme 2 (ACE2) receptor present on the cell surface of the human host. The ACE2 receptor is present in the respiratory organs, kidneys, gastrointestinal tract (at high levels in the esophagus, colon, and small intestine, but low in the stomach), and testes. Virulence of this novel virus is due to the presence of main protease responsible for virus replication along with many major functions ^6^. Therefore, we have employed the main protease structure 6m03 as the target protein to identify the best inhibitory drugs *in silico* for our study.

SARS-CoV-2 (negatively stained) when observed under the electron micrograph was found to be spherical in shape with some pleiomorphic characteristic. The epithelial sections of human airway when observed, viruses were found in membrane bound vesicles in cytoplasm along with inclusion bodies. The virions appear similar to solar corona due to 9- to 12-nm distinctive spikes and the virions are 60 to 140 nm in diameter. Thus, it was established due to these morphological characteristics that this virus belongs to the Coronaviridae family along with its genome having more than 85% identity with a bat SARS-like CoV (bat-SL-CoVZC45, MG772933.1) genome as previously assessed via genome sequencing ^6^. SARS-Cov-2 initially infects lower airways, binds to ACE2 receptor on cells activating immune cells, thus, inducing the secretion of inflammatory cytokines and chemokines in human pulmonary system ^4^. Most COVID-19 patients exhibit flu-like symptoms within a span of two weeks from the exposure to the virus whereas there have been a majority rise in the asymptomatic COVID-19 patients.

In this work, we have performed high throughput virtual screening since it is the fastest approach in finding the probable drug against the target. High-throughput virtual screening (HTVS) of two databases was carried out via PyRx (Python prescription) software, which uses dock, Vina and Autodock as the docking tool. Autodock itself uses MGLTools comprising of computer aided drug discovery (CADD) pipeline for high throughput virtual screening of large databases for probable hits as target drugs. HTVS enables docking of multiple ligands on a single protein. PyRx is a freely available HTVS software. Docking results are based on the identification of pose visually and quantitatively using a scoring algorithm. Docking calculates the free binding energy (∆G) between the ligands and the protein. The free binding energy, thus calculated, is fundamental to the formation of complex systems in biochemistry and molecular biology. Lower free binding energy corresponds to a more favorable ligand binding affinity between a receptor and a ligand ^7^.

## Methods

### Molecular docking

Molecular docking is a bioinformatics method that allows predicting the orientation of a molecule, when it is bounded to another molecule ^8, 9^. There are two main approaches for molecular docking. The first approach describes the protein and the ligand as complementary surfaces ^10^. The second approach simulates the docking process calculating the ligand protein interaction based on the free binding energy ∆G ^11^.

### Molecule selection

Selection of database and the COVID-19 main protease structure In this study, we have docked the X-ray crystal structure of main COVID protease protein (PDB ID: 6M03, resolution: 2 Å) with 129 molecules obtained from DrugBank and 992 molecules from the Zinc Natural Product database. The list of 129 molecules are provided along with the link for Zinc natural database in the *Extended data* ^12^. These 129 molecules chosen were either antimalarial, antiparasitic, antibiotics, or antivirals, since hydroxychloroquine, remdesivir and ivermectin are antimalarial, antiviral and antiparasitic drugs, respectively. The Zinc Natural Product Database was chosen since most of the drugs are natural derivatives used against various diseases at present and it is a freely available database. Similarity search could not be undertaken since there is no known drug to function 100% against this novel disease at time of publication.

### Processing of macromolecules and ligands

Docking requires processing of the macromolecules and the ligands ^13, 14^. Water molecules were removed, polar hydrogen bonds were inserted into the crystal structure of 6m03 and it was converted to PDBQT format using AutoDock version 4. The energy of all ligands were minimized and they were converted into PDBQT files using Open Babel version 2.2.3 in PyRX version 0.8 ^15^.

### Molecular docking process

The grid box was determined as center the coordinates X:12.2632, Y:12.3998, and Z:5.4737, while as dimension the coordinates X:29.9242, Y:64.1097, and Z:48.1126.

The docking was done using Vina version 2.0 in PyRX. After the run, the out files stored in the user folder where the path run was specified in the edit preference. These output files were stored in PDBQT files, each having nine poses. The autodock application file was launched which then showed the empty dashboard along with "File" on the left hand corner of the page. The out file models were loaded using the "read molecule" application from the selected out file folder. Different poses were analyzed in the AutoDock tool. The pose with the lowest binding energy in kcal/mol was selected for further analysis. The docked molecules were then further converted into PDB format in PyMol and their interaction was studied using the software Discovery Studio version 4.1. The interaction can also be studied with PyMol but a better quality picture is obtained via Discovery studio. Three known drugs (hydroxychloroquine, remdesivir and ivermectin) were first docked against the virus main protease to check their binding energy. The interaction of these three drugs with the COVID-19 main protease could later be utilised for getting the hits.

## Results

### Docking results for reference molecules

The free binding energy for drugs known to act against COVID-19, which are hydroxychloroquine, remdesivir, and ivermectin, were found to be -5.5 kcal/mol, -6.3 kcal/mol, and -8.7 kcal/mol respectively as indicated in Table 1, which describes: a) PubChem compound ID (CID), which is the compound identifier in the PubChem database from where the 3D mol files of 129 molecules were downloaded; b) common drug name; and c) the free binding energy obtained after docking.

These drugs are known to improve the condition to some extent and yet their functions against COVID-19 are under study ^16– 19^.

**Table 1.** Docking of known COVID-19 inhibitors.

PubChem CID	Drug Name	Binding energy kcal/mol
6321424	Ivermectin	-8.7
121304016	Remdisivir	-6.3
3652	Hydroxychloroquine	-5.5

**Figure 1.**
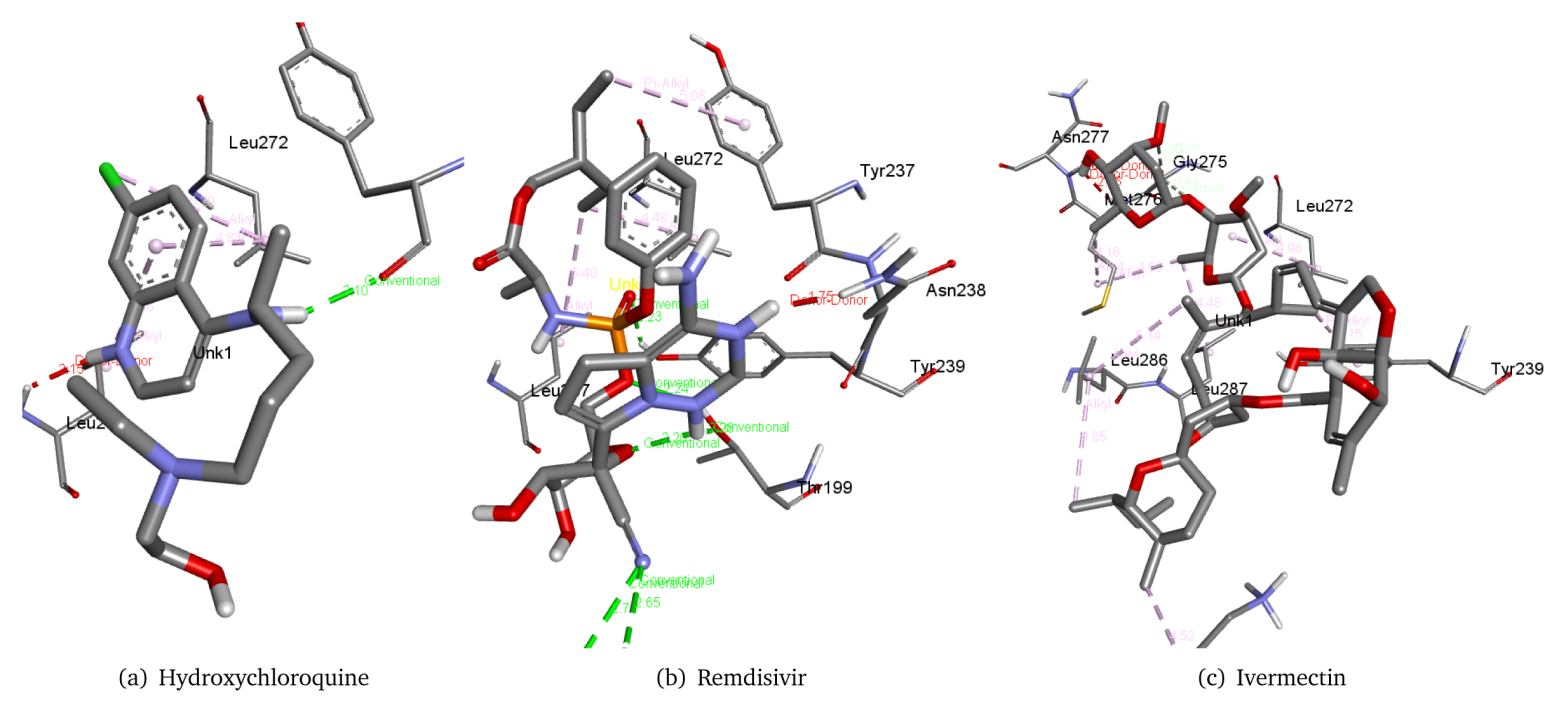
Reference molecules hydroxychloroquine, remdisivir, and ivermectin in complex with the COVID-19 main protease 6M03.

Therefore, we used these three molecules as our reference drugs. The interaction of these drugs with the virus main protease can be seen in the Figure 1. We could observe the interaction of these reference molecules as hydroxychloroquine forms a hydrogen bond with Tyr237 residue of the 6M03 main protease with a distance of 2.10 Å. It also interacts with Leu272 and Leu287. Remdisivir forms six hydrogen bonds with Lys137, Thr199, and Tyr239 along with interacting with Leu272, Leu287, Tyr237, and Asn238 residues of the 6M03 main protease. Ivermectin interacts with Leu272, Tyr239, Leu286, Leu287, Gly275, Asn277, and Met276 residues of the 6M03 main protease.

### Docking results for 129 additional molecules

**Figure 2.**
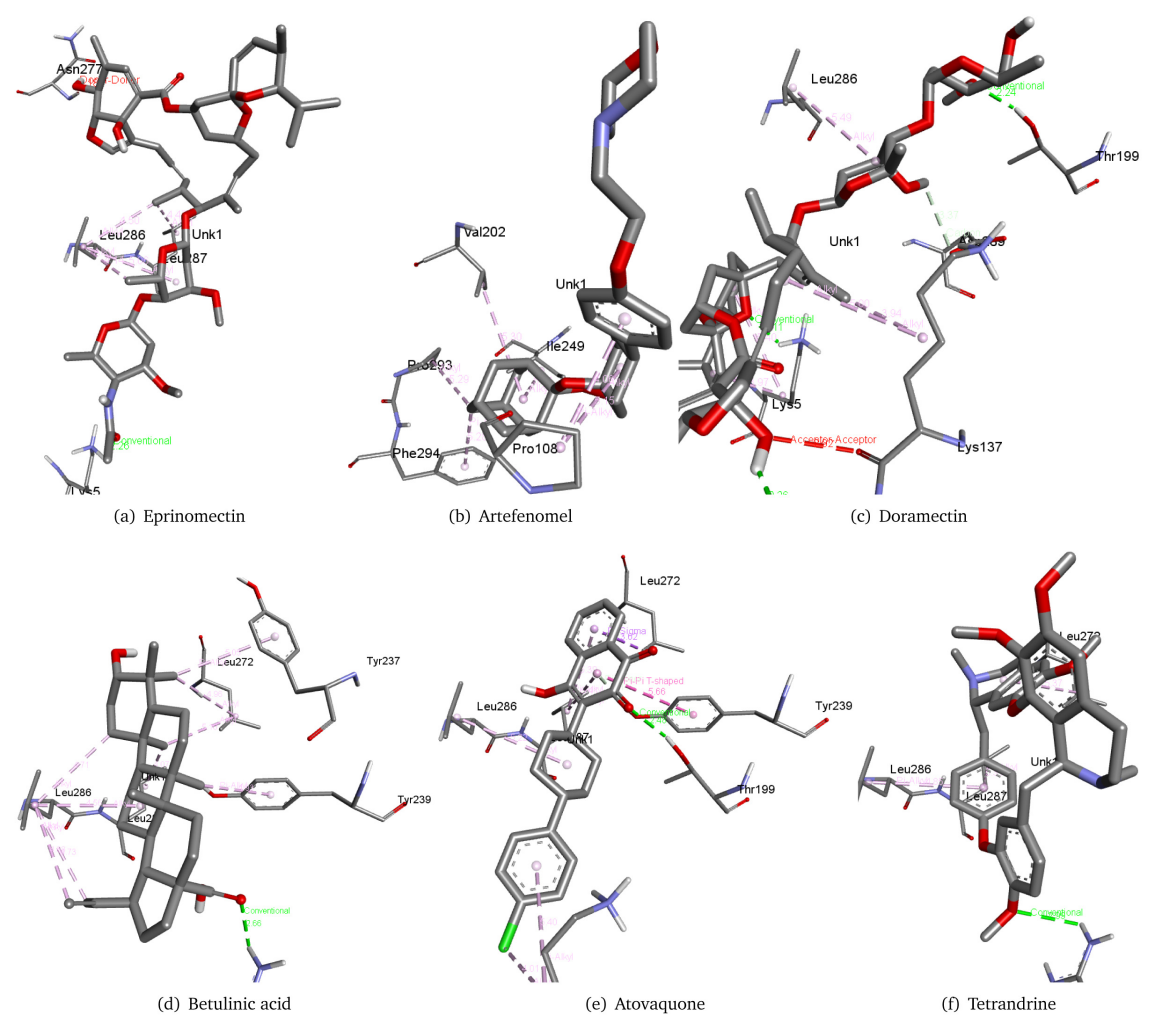
Interaction of COVID-19 main protease 6M03 with ligands eprinomectin, artefenomel, doramectin, betulinic, atovaquone, and tetrandrine.

Keeping the free binding energy of our reference molecules in mind, we shortlisted 77 molecules from the database of 129 molecules with a cut of -6 kcal/mol free binding energy. Eprinomectin, artefenomel, doramectin, betulinic acid, atovaquone, and tetrandrine showed the lowest binding energies, at -9 kcal/mol, -8.7 kcal/mol, -8.4 kcal/mol, -8.4 kcal/mol, -8.2 kcal/mol and -8 kcal/mol, respectively. Table 2 presents the details of the best performing 18 of the 77 molecules along with their CID. These molecules have been considered due to the lowest free binding energy between the ligand and protein. Furthermore, their interactions with the virus main protease 6m03 was studied. Artefenomel interacts with Pro108, Val202, Ile249, Pro293, and Phe294 residues of the 6M03 main protease. Eprinomectin forms a hydrogen bond (2.26 Å) with Lys5 residue of the 6M03 main protease and also interacts with Leu286, Leu287, and Asn277. Tetrandrine forms a hydrogen bond with Arg131 and also interacts with Leu272, Leu286 and Leu287. Betulinic acid forms a hydrogen bond with Arg137 with 2.66 Å distance and also interacts with Leu272, Leu286, Leu287, Tyr237, and Tyr239. Doramectin forms three hydrogen bonds with Thr199, Lys5, and Gly138 as well as interacts with Leu286 and Asp289. Atovoquone forms a hydrogen bond with the Thr199 residue of the 6M03 main protease with a distance of 2.48 Å and also interacts with Lys137, Leu272, Leu286, Leu287, and Tyr239. These interactions can be observed in Figure 2. Full results are available in the *Extended data* ^12^.

**Table 2.** Docking of drugs for probable COVID-19 inhibition.

	PubChem CID	Drug Name	Binding Energy kcal/mol
1	6450531	Eprinomectin	-9
2	24999143	Artefenomel	-8.7
3	9832750	Doramectin	-8.4
4	64971	Betulinic acid	-8.4
5	74989	Atovaquone	-8.2
6	73078	Tetrandrine	-8
7	6918632	Emodepside	-7.9
8	124081896	Baloxavir marboxil	-7.9
9	122262	Piperaquine	-7.9
10	35802	Flubendazole	-7.8
11	6917864	Artesunate	-7.6
12	40692	Mefloquine	-7.6
13	4030	Mebendazole	-7.6
14	21389	Imidocarb	-7.6
15	9832912	Moxidectin	-7.5
16	439530	Puromycin	-7.5
17	6323491	Radicicol	-7.4
18	54671203	Doxycycline	-7.4
19	107771	Pyronaridine	-7.4
20	11979956	Hachimycin	-7.3

### Docking Results for natural molecules

**Figure 3.**
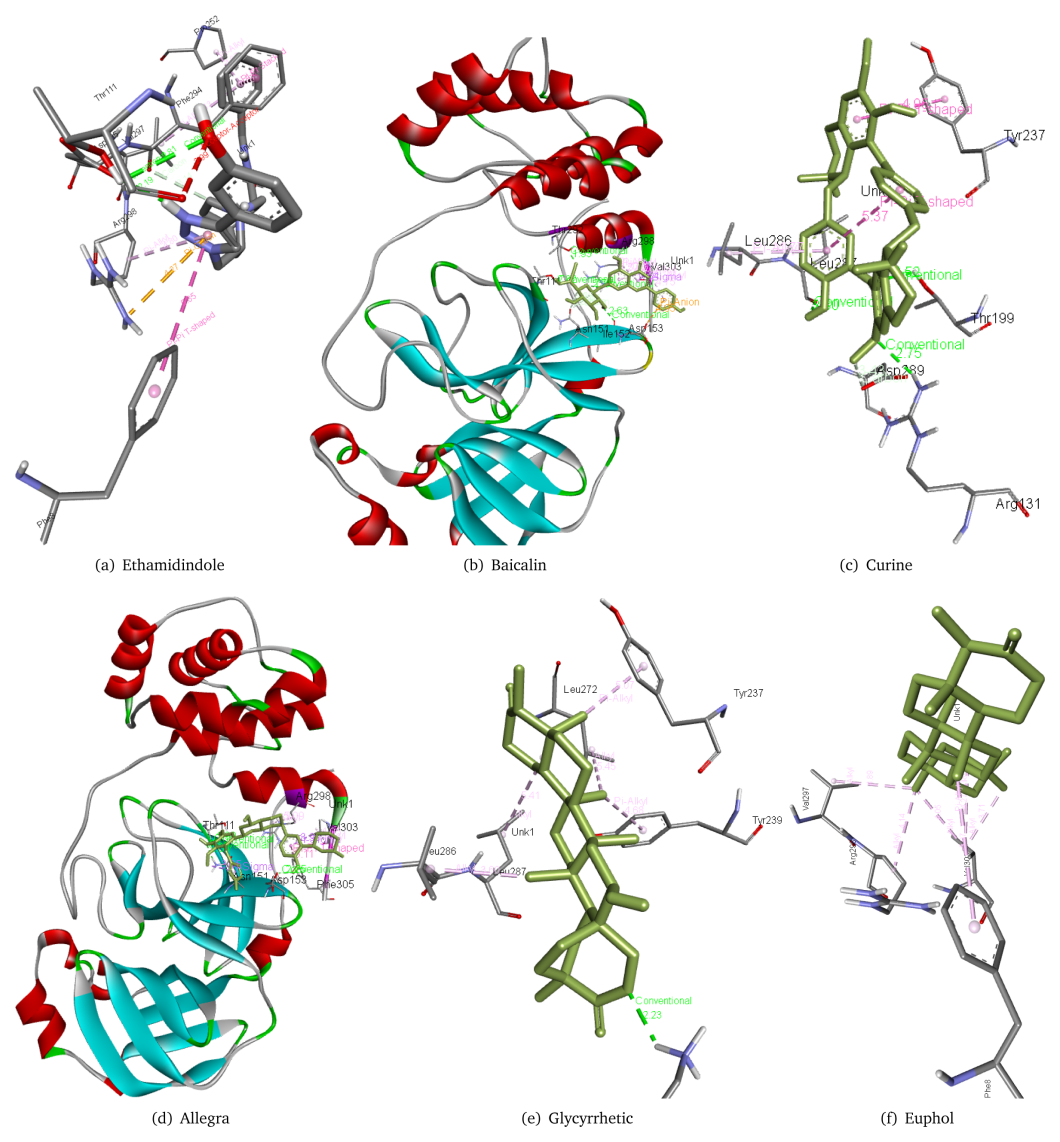
Interaction of six of the top 20 hits from Zinc natural database with main protease 6m03.

A total of 34 molecules of natural origin were chosen from the datasets of 992 molecules with a cut off -8.9 kcal/mol. Table 3 presents the details of the best 20 molecules along with their common name and ZINC ID sorted by ascending order of the free binding energy of the top 20 molecules from natural products database. The interaction of various molecules with the 6M03 main protease was studied. Allegra forms three hydrogen bonds with Thr111, Asn151, and Asp153 as well as interacts with Arg298, Val305, and Phe305 residues of 6M03 main protease. Baicalin forms four hydrogen bonds with Thr111, Thr292, Ile152, and Arg298 along with interacting with Asp153, Asn151, and Val303 residues of the main protease 6M03. Curine forms three hydrogen bonds with Arg131, Thr199, and Leu287 along with having interactions with Asp289, Leu286, and Tyr237 residues of 6m03 main protease. Etamidindole forms two hydrogen bonds with Thr111 and Asp295, it also interacts with Phe8, Phe294, Arg295, Arg298, and Pro252 residues of main protease 6M03. Glycyrrhetic acid forms a hydrogen bond with Lys137(2.24 Å) as well as interacts with Tyr237, Tyr239, Leu272, Leu286, and Leu287 residues of the main protease 6m03. Euphol interacts with Phe8, Val297, Arg298, and Val303 residues of the main protease. The interaction of few of the 20 molecules with the protein can be seen in Figure 3. Full results are available in the *Extended data* ^12^.

**Table 3.** Docking of natural compounds for probable COVID-19 inhibitory activity.

	ZINC *Prefix: ZINC000* ID	Common name	Binding energy kcal/mol
1	085592428	Furobinordentatin	-9.9
2	085592420	Alstiphyllanine F	-9.8
3	004104836	Taraxerone	-9.6
4	085593550	Morusin	-9.6
5	085596349	Fexofenadine acetate (Allegra)	-9.6
6	085648318	RA VII compound	-9.4
7	253589870	Neoruscogenin	-9.3
8	006627242	Justicidin D / neojusticidin A	-9.3
9	005742262	Licoricidin	-9.2
10	085643829	Euphol	-9.2
11	085593537	Schisandrene	-9.2
12	029483258	Curine	-9.1
13	005808583	Bikaverin	-9.1
14	014966151	Angoluvarin	-9.1
15	013543704	Baicalin	-9.1
16	085642858	Glycyrrhetic acid	-9.1
17	004995171	Isomitraphylline	-9
18	008829747	Rutarensin	-8.9
19	034075173	Jolkinol B	-8.9
20	003838672	Ethamidindole	-8.9

## Discussion

HTVS is one of the best methods for identifying molecules acting against drug targets in a very short time period, when compared to traditional drug identification strategies. Remdesivir was detected as COVID-19 inhibitory drug via virtual screening method ^6^. Therefore, keeping the present scenario in our mind, we undertook this study to provide individuals with COVID-19 with drugs already available. Surprisingly, we have found good hits from the databases with medicinal properties.

Artefenomel is known to treat malaria and other parasitic diseases. Betulinic acid is under trial for the treatment of dysplastic nevus syndrome. Atovaquone is an approved drug for the treatment of *Pneumocystis carinii* pneumonia and malaria. Tetrandrine is in the experimental stage for anticancer, antimalarial, antiparasitic category. Eprinomectin and doramectin are veterinary antiparasitic drugs.

Many of the natural compounds identified have medicinal properties. Taraxerone has allelopathic and antifungal effect ^20^, Morusin has anti-oxidant and anticancer properties ^21^, RA VII compound is an antitumor agent ^22^, and neoruscogenin is used against chronic venous disorders ^23^. Justicidin D exhibits anti-inflammatory properties ^24^, Licoricidin is an antimetastatic molecule ^25^ whereas euphol is used against asthma and cancer along with syphilis, and rheumatism ^26^. Schisandrene has anti-oxidant activity ^27^, curine is reported as a vasodilator ^27^, angoluvarin has antimicrobial activity ^28^, baicalin is used to treat cardiovascular diseases, inflammation and hypertension ^29^. Glycyrrhetic acid shows anti-inflammatory, anti-ulcer, hepatoprotective, anti-allergic, anti-tumor, antioxidant and anti-diabetic activity ^30^. Isomitraphylline has an antioxidant properties ^31^, bikaverin and rutarensin has anti-tumour activities ^32, 33^. Jolkinol B has anticancer properties ^34^ and Ethamidindole exhibits antihistamine properties ^35^. Fexofenadine acetate, commonly known as Allegra, is an antihistamine pharmaceutical drug presently used in the treatment of allergy symptoms such as urticaria and hay fever ^36^.

When we compared our two datasets, we found that majority of the molecules showed lesser free binding energy as compared to the reference molecules as in Table 1, Table 2, and Table 3. Surprisingly, we obtained good hits from our natural database which is good news since after observing their interaction and biotherapeutic functions, we might have achieved our COVID-19 inhibitory drugs. One such drug from natural database is fexofenadine acetate (Allegra) which is presently in use as anti-allergic medicine. We also have hits from 129 drugs but there is one, atovaquone, which is presently used against pneumonia and malaria and also a very good candidate for COVID-19 treatment.

## Conclusions

The best therapeutic drugs inferred from our studies are atovaquone, fexofenadine acetate (Allegra), justicidin D, baicalin, glycyrrhetic acid and ethamidindole based on their docking score, interaction studies and their present applications for probable COVID-19 treatment. The rest of the molecules could also be used as COVID-19 inhibitory drugs after further evaluation. When we compared our data with reference molecules score of currently in use drug against COVID-19, we found that atovaquone showed better binding energy than hydroxychloroquine and remdesivir. It is one of the best drug candidate for COVID-19 treatment since it is already in use for treating *Pneumocystis carinii* pneumonia and malaria. Fexofenadine acetate is another good target drug for COVID-19 treatment since it is naturally derived and presently used for its anti-histamine properties. Ethamidindole could possibly act as COVID-19 inhibitor since it is reported as anti-histamine and this novel virus activates cytokine secretion in human body. Apart from these, anti-inflammatory natural molecules such as justicidin D, baicalin, and glycyrrhetic acid could work against COVID-19 since SARS-CoV-2 virus induces inflammation. The rest of the top 20 molecules could also be considered since they all have some medicinal properties as explained above.

## Data availability

### Source data

The COVID-19 main protease structure was downloaded from the Protein Data Bank, ID 6M03: https://www.rcsb.org/structure/6M03.

Ligands were obtained from PubChem ( https://pubchem.ncbi.nlm.nih.gov/) and the Zinc Natural Products database ( http://zinc.docking.org/).

### Extended data

Zenodo: Molecular docking COVID-19. https://doi.org/10.5281/zenodo.3840625 ^12^.

This project contains the following extended data:

- Drugs repurposing list (PDF). (PubChem CID of each ligand along with the minimized energy of each molecule and binding affinity results)
- Drugs (subfolder). (Interaction images of the reference molecules as well as the best performing target molecules from the 129 docked drugs.)
- Natural compounds (subfolder). (Interaction images of the best performing target molecules from the Zinc Natural Products database.)
- Results (PDF). (Complete docking results from the Zinc Natural Products database and the list of 129 drugs.)

Extended data are available under the terms of the Creative Commons Attribution 4.0 International license (CC-BY 4.0).
