# Supplementary material for: Coronavirus disease 2019 drug discovery through molecular docking
Source: F1000Res. 2020 Jun 3;9:502. [Version 1] doi: 10.12688/f1000research.24218.1 (PMC7361499; doi:10.12688/f1000research.24218.1)
Supplement: Peer review history

# Peer review: Reviewer response for version 1

**DOI:** 10.5256/f1000research.26715.r67037

**Published:** 2020-07-14

**Authors:** Pritha Ghosh

**Affiliations:** 1 Laboratory of Bioinformatics and Protein Engineering, International Institute of Molecular and Cell Biology in Warsaw, Warsaw, 02-109, Poland

The manuscript by Singh and Florez presents the discovery of drugs against COVID19 using molecular docking methods.

**Major:**

1. The authors must discuss in further detail as to how this work compares with other published reports of drugs targeted against SARS-CoV-2.
2. The authors should cite references for the results mentioned in the Introduction section of the manuscript.
3. How was the -6 kcal/mol free binding energy cut-off selected? As I understand this is not the lowest, the highest or the average of the reference molecule binding energies. Then what is the basis for the selection of this cut-off value?
4. The authors have discussed the docking results for the reference and the test molecules independently, but have not provided a comprehensive comparison of the observations. In my opinion, such a comparison is necessary for assessing the effectivity of the new drugs as compared to the existing drugs. The authors have also not discussed the importance of the residues Tyr237, Leu272, Leu287 etc. Are these known to be critical residues in the functioning of the main protease?

**Minor:**

1. The statistics on the number of cases and deaths need to be updated before the indexing.
2. The authors should be more careful about the technicalities mentioned in the paper. For example, "6m03" has been mentioned loosely in the Introduction section without pointing to the fact that it is a PDB structure; the HTVS abbreviation has not been introduced in the manuscript before the first instance of its use.

The paper requires English editing by a professional and/or a native English speaker.

Is the work clearly and accurately presented and does it cite the current literature?

Partly

If applicable, is the statistical analysis and its interpretation appropriate?

Not applicable

Are all the source data underlying the results available to ensure full reproducibility?

Yes

Is the study design appropriate and is the work technically sound?

Partly

Are the conclusions drawn adequately supported by the results?

Partly

Are sufficient details of methods and analysis provided to allow replication by others?

Yes

Reviewer Expertise:

Bioinformatics and Genomics

I confirm that I have read this submission and believe that I have an appropriate level of expertise to confirm that it is of an acceptable scientific standard, however I have significant reservations, as outlined above.

# Peer review: Reviewer response for version 1

**DOI:** 10.5256/f1000research.26715.r64264

**Published:** 2020-07-06

**Authors:** Fernando Yepes-Calderon

**Affiliations:** 1 Division of Neurosurgery, Children's Hospital Los Angeles, Los Angeles, CA, USA; 2 SBP LLC, Reserach and Development, Fort Pierce, FL, USA

The paper is of interest under the current cov2 pandemic. Simulation of the strength of the bonds between molecules can identify inhibitors without human subjects' need to test. This paper did not focus on finding new compounds but in examining those present in existing medicine. This approach expedites the process of finding a cure, and the suggestions done are well-founded. Overall, the materials and methods section is exhaustive and asserts reproducibility.

 There are some wordy paragraphs with redundancies. Please take a look at the following one and the replacement that I suggest.

 Original> The transmission of this coronavirus occurs due to the binding of the CoV spike protein to the angiotensin converting enzyme 2 (ACE2) receptor present on the cell surface of the human host.

 Suggestion>The transmission of this coronavirus occurs due to the binding of the CoV spike protein to the angiotensin-converting enzyme 2 (ACE2) receptor present on the human host's cell surface.

 I might suggest the authors remark the differences  between their approach and the one presented by:

 Suyash Pant, Peptide-like and small-molecule inhibitors against Covid-19 ^1^

 It could be included in the discussion.

Is the work clearly and accurately presented and does it cite the current literature?

Partly

If applicable, is the statistical analysis and its interpretation appropriate?

Not applicable

Are all the source data underlying the results available to ensure full reproducibility?

Yes

Is the study design appropriate and is the work technically sound?

Yes

Are the conclusions drawn adequately supported by the results?

Yes

Are sufficient details of methods and analysis provided to allow replication by others?

Yes

Reviewer Expertise:

Methods in medicine, Medical Imaging, Artificial Intelligence

I confirm that I have read this submission and believe that I have an appropriate level of expertise to confirm that it is of an acceptable scientific standard.

## References

[1] : Peptide-like and small-molecule inhibitors against Covid-19. *J Biomol Struct Dyn*.2020; 10.1080/07391102.2020.1757510 1-10 10.1080/07391102.2020.1757510 32306822PMC7212534

# Peer review: Reviewer response for version 1

**DOI:** 10.5256/f1000research.26715.r64263

**Published:** 2020-06-29

**Authors:** Elio Rodrigo Castillo

**Affiliations:** 1 Laboratorio de Genética Evolutiva, Instituto de Biología Subtropical (IBS), FCEQyN, CONICET-UNaM, Posadas, Argentina

I think that all the data is a quite good contribution, but in my opinion, text should be deeply modified to be more attractive to the readers and to point more directly their main objective.

**Introduction**

**Paragraph 2:** I suggest the authors to focus in the evidences showed by Chang *et al.* We know that vaccines are not available for COVID yet but your aim is to identify **potential** drugs for treatment.

**Paragraph 3:** Move this paragraph below “ *The World Health Organization (WHO) declared this disease as a pandemic on 11th March, 2020 and SARS-CoV-2 as the deadliest virus till date on earth claiming 217,896 deaths till 30th April, 2020”.*

**Paragraph 4:** Please remove this paragraph because is not discussed in the corresponding section.

**Paragraph 5: **I recommend the authors to rewrite this paragraph. Authors performed an approach for searching drugs candidates based on databases. Please, focus in the main objective of the work. In my opinion authors could highlight the importance of bioinformatic tools (docking as the first approach) using available databases. I suggest to rewrite this paragraph because most of it content correspond to the Methodology section.

**Methods**

 In my opinion authors should be more clear with the methodology. You could cite a work in the introduction section where docking was the better strategy for drugs candidates search. In this section, you should explain your criteria of protein selection, provide a list and finally, the docking process. I suggest to reorganize this section as:

 "Molecule selection and processing (or edition)" and “Molecular docking”. In my opinion it is not necessary to split this information.

**Results**

 For a better understanding, I strongly recommend to use a general graphical scheme explaining the selection, the macromolecules edition, and the pipe used for the docking analysis. 

 I suggest to focus only in the docking results against different ligands (from databases, natural etc). Some sentences correspond to the discussion section.

**Discussion**

 It is not clear if you work with information from databases or with clinical samples. Please, rewrite this sentence.

 The paragraph underline (2nd) is disconnected from this section, please review.

 In my opinion the third paragraph of this section is not necessary. Authors could write in a sentence all this information and cite.

 I also suggest to discuss better your results and make a contrast with the available bibliography; authors repeated in this section the results. I understand that your contribution is relevant but from other perspective: bioinformatic tools for a quick detection of potential antivirals or drugs, using as an example the COVID.

 Several papers cited in the introduction section are not discussed.

**Conclusions**

 I suggest the integration of this section with the discussion. Authors proposed several treatments, inferred from HTVS, however, you should be careful with your statements. I recommend to discuss and compare the variables or data as indicators for potential treatments.

**The manuscript is written with understandable English, though some English revision is necessary.**

**In my opinion, after several major revisions and reorganization of the text, the study will be acceptable for indexing on F1000Research.**

Is the work clearly and accurately presented and does it cite the current literature?

Partly

If applicable, is the statistical analysis and its interpretation appropriate?

I cannot comment. A qualified statistician is required.

Are all the source data underlying the results available to ensure full reproducibility?

Yes

Is the study design appropriate and is the work technically sound?

Yes

Are the conclusions drawn adequately supported by the results?

Partly

Are sufficient details of methods and analysis provided to allow replication by others?

Yes

Reviewer Expertise:

Evolutionary Biology, Genomics

I confirm that I have read this submission and believe that I have an appropriate level of expertise to confirm that it is of an acceptable scientific standard, however I have significant reservations, as outlined above.
